# Motivation and behavioral mechanisms of physical activity in community-dwelling elderly populations

**DOI:** 10.3389/fpsyg.2026.1767861

**Published:** 2026-04-10

**Authors:** Jian Wei, Huiguo Wang

**Affiliations:** 1 Department of Physical Education, School of Physical Education, Northeast Normal University, Changchun, Jilin, China; 2 School of Sport and Health Sciences, Guangzhou Sport University, Guangzhou, Guangdong, China

**Keywords:** capability, COM-B model, English Longitudinal Study of Ageing, environmental opportunity, motivation, older adults, physical activity, self-determination theory

## Abstract

**Introduction:**

Physical activity (PA) is essential for healthy aging, yet participation declines as older adults face interacting social, environmental, motivational, and functional barriers. Existing research rarely integrates these determinants to explain how context and capability shape PA behavior.

**Methods:**

This study examined the mechanisms linking environmental opportunity, social support, motivation, and capability to PA among community-dwelling older adults using an integrated framework grounded in Self-Determination Theory (SDT), the COM-B model, and social-ecological principles. The English Longitudinal Study of Ageing (ELSA) was an adult study (3,842 adults aged 60 years and above) that collected data. Interventions were PA, social support, community involvement, environmental opportunity, motivational constructs (autonomy, competence, self-efficacy, identity), and functional capability. Descriptive statistics, correlation analysis, and structural equation modeling (SEM) were performed, along with tests of mediation and moderation.

**Results:**

The most significant predictors of moderate and vigorous PA were motivation. The effects of environmental opportunity and social support were mainly indirect, through motivation, and the motivation pathways explained 56–63% of the overall impact. The relationships were strongly moderated by capability: the motivational effects were more pronounced among those with fewer functional limitations. The sensitivity analysis proved the soundness of the integrated model.

**Discussion:**

PA in old age represents the product of an active interaction between motivational, social, environmental, and capability-related aspects. The core route from external conditions to behavior comprises motivational processes, and the magnitude of motivation to act is determined by capability. Motivational strengthening, the supportive environment, and customized capability plans should be combined into effective interventions to facilitate active aging.

## 1 Introduction

Regular physical activity (PA) among the elderly is commonly considered an ingredient of healthy aging: it can sustain functional ability and mobility, reduce the risk of chronic illnesses (e.g., cardiovascular disease, diabetes), slow or prevent frailty, and improve mental health and independence in everyday life. Moreover, definitions and categorization of PA across studies were quite different, which diminished the comparability and interpretability (D’Amore et al., 2023). In addition, many intervention studies conducted to increase PA among older adults are poorly conceptualized with respect to their theoretical foundation (e.g., motivational, identity, habit theories) and do not investigate why and whom interventions benefit in the long term. This indicates a larger gap: inadequate comprehensive models have been developed to combine intrapersonal (e.g., motivation, self-efficacy), inter-personal (e.g., social support), and environmental (e.g., neighborhood design, built environment) determinants, and there are also no empirical tests of the interaction between these levels to determine the effects of such levels on PA behavior over time. Therefore, despite decades of study, the discipline is deficient in both mechanism-based and longitudinal, as well as theory-driven analyses of large, representative cohorts of older adults. In the absence of such studies, there are few opportunities to develop sustainable, contextually-based PA interventions. The increased focus on social and environmental determinants of PA behavior among older adults has gained attention in recent years. It has been indicated that social support, through encouragement from family members or friends, companionship, everyday activities, and broader contact with society, assists in the establishment and maintenance of PA among older adults. A recent scoping review found that there is a strong correlation between functional social support and PA in older adults (mean age 60 years and above), with more substantial social support associated with more frequent PA (Lindsay Smith et al., 2017). Qualitative and mixed-methods studies also highlight the significance of companionship and shared social purpose in motivating older adults to remain active (Meredith et al., 2023). Environmental conditions, particularly the qualities of the built and neighborhood environment like walkability, proximity of public spaces, safety, availability of green space, and pedestrian infrastructure, also have significant impacts on the participation of older adults in PA. A number of systematic reviews and empirical studies show that the positive relationship between neighborhood walkability and supportive built environment characteristics and walking and other types of PA among older adults is present (Cleland et al., 2019; Bringolf-Isler et al., 2019). An example of this is the fact that research has indicated that elderly individuals living in neighborhoods where walking is possible tend to indulge in more regular walking, either to get to their destinations or as a way of recreation, compared to those in areas that are not very walkable (Van Holle et al., 2014). The results are, however, not consistently uniform. Other reviews show numerous null associations between built environment characteristics and PA in older adults (D’Amore et al., 2023), and that the bulk of existing research investigates environmental variables only and fails to include social and psychological moderators. It means that the built environment is not a panacea, yet it can work alongside social support, personal motivation, self-efficacy, and identity. This leads to the conclusion that social, environmental, and psychological determinants should be analyzed in a more theory-informed manner to gain a more insightful view of PA behavior in old age. To address the theoretical and empirical gaps presented, the data collected on the ELSA will be used in this research. ELSA is a nationally representative, longitudinal cohort study of the 50-plus population in England that gathers rich, multidimensional data on health, socioeconomic status, social participation, lifestyle behaviors (including PA), psychosocial factors, and environmental conditions (Bonaccorsi et al., 2020). Accordingly, the present study aims to:

- Test a comprehensive, integrated behavioral model that links environmental opportunity, social support/community engagement, motivational and self-efficacy constructs, and PA behavior in community-dwelling older adults.
- Examine whether these relationships vary by health-related capability, while also describing age-group differences in PA patterns.
- Provide empirical evidence to inform community and environmental interventions designed to promote PA among older adults.

By doing so, this paper contributes to the special issue on “Motivations for PA” by offering a mechanism-based, contextually grounded, and policy-relevant analysis of PA behavior in older age, advancing beyond descriptive correlates toward actionable theory and intervention design.

The paper is structured as follows. Section 2 is a review of empirical studies conducted on motivational, social, and environmental predictors of PA in older adults. Section 3 describes the synthesized theoretical model that entails the SDT, the COM-B model, and social-ecological factors. In Section 4, the data, measures, and method of analysis will be outlined: the ELSA and SEM. Section 5 presents the empirical findings, including pattern descriptions, correlations, and conclusions from mediation and moderation analyses. Section 6 explains the implications of the results for the theory and past studies, and Section 7 provides the conclusion regarding the main impact of the results on policy, practice, and future research.

## 2 Literature review

### 2.1 Motivational determinants in older adults

The lifespan determinant of PA is motivation. Nonetheless, it is essential during the elderly adulthood as the functional capacity, overall health, perceived abilities, and daily activities change. Literature based on SDT suggests that older adults who have greater autonomy, competence, and relatedness tend to remain more PA behavioral in the long-term (Ahmed et al., 2020). Positive affect and enjoyableness, as intrinsic motivation factors, as well as perceived well-being, have been associated with long-term PA adherence among the elderly (Palombi et al., 2025; Stevens et al., 2020). Self-efficacy continues to be among the most effective predictors of PA among aging populations, influencing the formation of intentions, perceived capability, and the overcoming of mobility barriers (Xie et al., 2025). The meta-analysis evidence has shown that self-efficacy is a predictor of PA initiation and maintenance in adults 60 years and older (Szczuka et al., 2021). The identity theory is also a factor: an active identity allows older adults to internalize PA into their self-concept, which ensures that they remain more active (Beauchamp et al., 2018). On the other hand, age-related negative stereotypes (e.g., frailty or decay) could reduce intrinsic motivation to participate in PA due to reduced perceived competence and threat appraisal (Fougner et al., 2019). Numerous studies indicate that motivational deterioration in later years is associated with health worries, concerns about harm, incapacity, and functional constraints, which tend to decrease self-reliance and perceived ability (Jofré-Saldía et al., 2023). All in all, the literature indicates that the motivational determinants are multi-dimensional and involve the interplay among internal (self-efficacy, intrinsic motivation), emotional (enjoyment, affect), and identity factors. Nevertheless, such motivational channels are seldom discussed in relation to social or environmental factors.

### 2.2 Social support, community engagement, and PA

One of the strongest, but intermittently measured, determinants of PA in older adults is the social factors. It has been demonstrated in various studies that social support from family, peer, and community networks can help with PA participation through encouragement, companionship, and accountability (Van Luchene and Delens, 2021). A recent scoping review of older adults living in the community found robust associations between functional social support (emotional and instrumental) and PA engagement (Ferreira et al., 2024). The involvement of the community, club membership, volunteering, and working in groups are other issues that have become stable predictors of PA sustainability and serve as both motivational and behavioral reinforcers (Carr et al., 2018). The elderly who belong to social groups are more likely to have high levels of PA, because of the possibility to engage in activity together, the power of social norms, and less isolation (Delgado et al., 2023). Social connectedness has also been observed to mediate the adverse consequences of health deterioration against PA levels, strengthening the role of interpersonal affiliations (Anyan et al., 2020). Interventions conducted in groups are especially effective for older adults, as peer support has been shown to enhance compliance, enjoyment, and perceived value of PA programs (Aprobato et al., 2024; Farrance et al., 2016). There are also social network communities that have been linked to walking frequency and active commuting patterns among the elderly, such as social network size, density, and supportive ties (Asiamah et al., 2020). Social support is not fixed, however. Social networks may be undermined by bereavement, retirement transitions, and relocation, which in turn diminishes PA behavior (Baxter et al., 2016). Therefore, life-course changes and interpersonal situations should be incorporated into behavioral models better to understand the effects of social dynamics on PA.

### 2.3 Environmental opportunity and neighborhood context

The environmental opportunity is vital in determining the PA behaviors of older adults because environmental barriers are likely to become more constraining as mobility declines. Positive correlations between PA levels in aging populations and walkability, access to parks, safety, pedestrian infrastructure, and perceived neighborhood aesthetics have been reported (Kotlarczyk et al., 2020). Systematic reviews show that older adults who reside in walkable, mixed-use areas tend to walk more daily, engage in recreational PA, and adhere to PA guidelines (Fisher et al., 2018). The characteristics that have been found to enhance outdoor walking and park use among older adults include neighborhood safety, such as low traffic, good lighting, and lower crime rates (Blackwood et al., 2022). Equally, the availability of green spaces and open areas promotes leisure walking and moderate-intensity PA, especially in chronic residents (Kou et al., 2021). Poor transportation facilities, uneven pavements, and steep slopes in the environment have been cited as formidable deterrents to PA (Choi and DiNitto, 2016). However, the results of environmental determinants are inconclusive. Several large-scale reviews have observed a high proportion of non-significant associations between built-environment characteristics and PA in older adults (D’Amore et al., 2023; Thornton et al., 2017). These discrepancies underscore the need to consider integrative models that explain the interrelations among the environment, social context, and psychological determinants.

### 2.4 Community-based program evidence

It has been demonstrated that community-based PA programs can enhance functional health, increase activity levels, and improve the quality of life for older adults. There is some evidence that a structured group intervention, including community walking groups, senior physical exercise classes, strength and balance training programs, and volunteer groups, can be effective in achieving considerable improvements in PA compliance and functional outcomes (Vseteckova et al., 2020). Social programs, regular routines, and community involvement demonstrate increased participation and reduced dropout rates among the elderly (Lemieux et al., 2020). A systematic review found that community-based exercise programs reduced walking speed, improved balance and mobility, and lowered the risk of falls (Montero-Odasso et al., 2021). Multi-component interventions that combine behavioral counseling with social support or environmental changes have more lasting effects than single-focus interventions (Kwok et al., 2024). There is also some promise in technology-based community interventions (e.g., exergames, virtual courses) that would be especially helpful for older adults with mobility limitations, but digital literacy is a barrier to implementation (Ferguson et al., 2022). Nevertheless, most community programs are poorly theorized or fail to analyze the processes underlying their success, thereby limiting their external validity (Janols et al., 2022). The access to the programs is also not always equitable: the socially isolated, economically disadvantaged, and low-resource elderly people are much less likely to be involved in the community programs (Coll-Planas et al., 2018).

### 2.5 Limitations in previous mechanistic research

In past studies, one consistent issue has been the absence of a mechanistic focus. Numerous studies have found correlations but have not validated the interaction among motivational, social, and environmental pathways in influencing PA behavior. As observed in several reviews, cross-sectional designs are commonly used in studies, which limit the causal interpretation and mask dynamic changes in PA behavior (D’Amore et al., 2023; Marques et al., 2019). In the absence of longitudinal data, the study will not be able to adequately measure habit formation, changes in motivations, or the influence of social and environmental changes. Moreover, in most studies, determinants are examined in isolation, e.g., motivation alone or social support alone, rather than within the context of more comprehensive behavioral models, e.g., SDT, COM-B, or ecological models (Lobczowska et al., 2022). Such disintegration complicates the development of concerted intervention plans that would respond to dissimilar degrees simultaneously. Mechanistic insight is also undermined by issues of measurement. Self-reported PA is utilized in the majority of the research, which is susceptible to recall bias and inaccuracy, especially in older adults who have depleted their cognitive functions (Sattler et al., 2021). The environment is frequently viewed through the prism of the neighborhood exposure without taking into consideration the micro-environmental variables (including the housing status and the walkability of the block), which can be more vital to the elderly (Abe et al., 2021). Social things (e.g., support, engagement) are crudely measured and do not reflect network dynamics, tie strength, or quality of interaction. Lastly, motivational constructs (e.g., self-efficacy, identity, intrinsic motivation) are also not adequately represented or consistently measured, so one cannot determine mediating pathways between the environment, social context, and PA (Friel and Garber, 2020). All these restrictions highlight the necessity of longitudinal, integrated, and mechanism-focused studies using a robust dataset such as ELSA.

## 3 Theoretical framework

**Figure 1:**
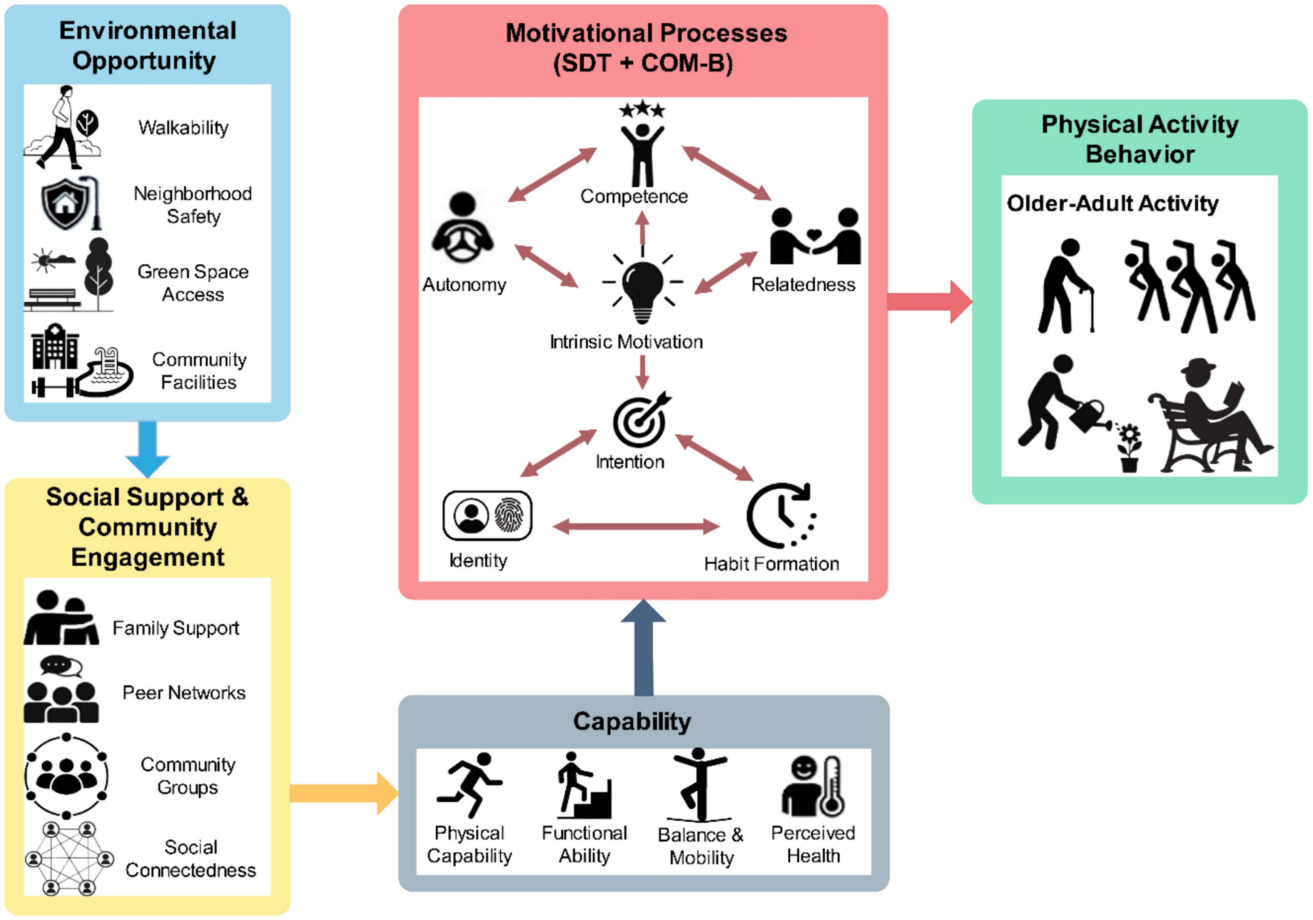
Conceptual framework integrating SDT, COM-B, and social-environmental determinants.

To obtain insights into PA behaviors among older adults living in communities, an integrative theoretical approach that explains behavioral, psychological, social, and environmental factors is necessary. The research is based on three complementary theories, namely SDT, the COM-B-behavioral model, and the Social-Ecological Model, to develop a conceptual framework that explains the initiation, persistence, or resignation to PA in older adults. The combination of these views is presented in Figure 1, which informs the hypotheses and empirical tests of this research.

### 3.1 Self-determination theory

SDT posits that the desire for autonomy, competence, and relatedness determines human behavior. These psychological needs are at the center of the internalization of health behaviors, such as PA. The feeling of autonomy, self-initiated choice of activities, facilitates pleasure and reduces unwillingness to participate among older adults. This competence (or successful and safe performance) is especially acute at old age, as functional capacity may vary. Relatedness, the concept represented by social connectedness and a sense of belonging, helps maintain emotional well-being and further engagement in activities. When these needs are fulfilled, the elderly will be more inclined to internalize PA as personally valuable, which results in intrinsic motivation and long-term adherence. On the other hand, motivation may be switched off whenever competence or relatedness is challenged by a sense of weakness, fear of harm, or even individual social isolation.

### 3.2 COM-B behavioral model

The COM-B model offers a contrasting view by focusing on how PA behavior is formed through the interaction among capabilities, opportunities, and motivation. The ability is determined by physical and psychological capacity to move, and, for older individuals, by mobility, chronic diseases, balance, confidence, or knowledge of an activity. Opportunity is used in reference to external provisions, the physical environment, and the social environment, which either make or inhibit activity. Motivation is both reflective, i.e., intentions and plans, and automatic, i.e., habits, impulses, and identity. These aspects are very intertwined within the context of aging. However, being inspired to act, older people may be unable to act due to an inability or the absence of a situation. At the same time, it can enhance motivation through conducive conditions and social systems that will affect habit formation. The concepts of COM-B take the form of mediating and moderating processes converting social and environmental factors into behavior.

### 3.3 Social-ecological influences on older adults

The Social-Ecological Model is an expansion of the behavioral approach, in that it demonstrates that the interplay of multiple levels of influence influences PA. Individual factors other than health, confidence, and attitudes depend on interpersonal factors, which are family encouragement, peer companionship, and social networks. The bigger picture of the activity is the community and environmental conditions: whether the neighborhood is walkable, whether there are green spaces, whether there is access to transportation, and whether the neighborhood is safe. Other channels through which older adults can engage in physical activities include organizational structures, such as senior centers and community groups, and the availability of programs. These circles of influence become increasingly relevant later in life, when day-to-day activities are closely linked to local settings, and the social network can be altered by pre-retirement, bereavement, or migration. The social-ecological perspective is reflected in its focus on external conditions of opportunity and on the interaction between these and individual motivation and capabilities to generate observed activity behavior.

### 3.4 Integrating SDT, COM-B, and environmental factors

Even though SDT, COM-B, and the Social-Ecological Model provide valuable insights, they are most effective when combined. The combined conceptual model incorporates psychological need satisfaction, behavioral processes, and multi-level contextual variables to describe the process by which older adults engage in and sustain PA. The opportunities of the activity and the significant motivational processes in this model depend on environmental and social conditions. Positive contexts could promote perceived competence, relatedness achieved through socialization, and autonomy, given the availability of a variety of activities. These psychological consequences support reflective and automatic motivation derivatives indicated in the COM-B model. This integration is dominated by capability. Behavior is limited by physical or functional constraints even in supportive environments, whereas available community design and safe spaces may only increase perceived and actual abilities. The model thus emphasizes that PA behavior does not arise solely from isolated effects but rather from the dynamic interaction of motivation, environmental opportunity, social conditions, and capability. In the present study, capability refers to the physical and functional capacity to engage in PA, consistent with the COM-B model, and is operationalized using indicators of functional limitations (ADL/IADL) and self-rated health/physical functioning.

### 3.5 Study hypotheses

The integrated theoretical model supports the development of the study’s hypotheses. It is expected that environmental opportunity, such as perceived neighborhood walkability and access to community resources, will be positively associated with PA among older adults.

*H1*: Environmental opportunity, such as neighborhood walkability, perceived safety, and access to community resources, will be positively associated with PA among older adults.

*H2*: Social support and community engagement will be positively associated with PA.

*H3*: Motivational factors (autonomy, competence, self-efficacy, enjoyment, active identity) will mediate the effects of:

*H3a*: Environmental opportunity for PA.

*H3b*: Social support/community engagement on PA.

*H4*: The relationships between environmental/social determinants and PA will be moderated by capability, which is the COM-B element that denotes physical/functional ability of activity and is assessed with the help of functional limitations and perceived health/physical functioning scales.

*H5*: The integrated model will demonstrate a strong overall fit in explaining PA behavior using SEM.

## 4 Data and methods

### 4.1 Dataset description: ELSA

The English Longitudinal Study of Ageing [English Longitudinal Study of Ageing (ELSA), 2024] is an ongoing, nationally representative cohort study designed to examine the health, economic, and social circumstances of adults aged 50 and over living in private households in England. The study collects detailed information on physical and psychosocial health, social participation, community involvement, lifestyle behaviors, and environmental perceptions. For this study, data from Waves 7 and 8 were used to capture a comprehensive set of variables related to PA, social determinants, environmental opportunity, and motivational factors. ELSA’s multi-dimensional nature makes it well-suited for testing the integrated conceptual framework described in Figure 1. The richness of measures allows for operationalization of constructs from SDT, the COM-B model, and the Social-Ecological Model within a single dataset. Descriptive characteristics of the final analytic sample are presented in Table 1, showing the distribution of demographic, health, social, and PA indicators among community-dwelling older adults.

**Table 1:** Sample characteristics of community-dwelling older adults (*N* = 3,842).

Characteristic	Mean/%	SD	Definition
Age (years)	71.8	7.4	Continuous age at interview
Female (%)	56.3	—	Sex assigned at birth
Married/Cohabiting (%)	64.1	—	Partnership status
Ethnic minority (%)	6.4	—	Self-identified ethnicity
Educational attainment (years)	11.2	3.1	Formal education duration
Self-rated health (1 = poor, 5 = excellent)	3.15	0.94	General health status
Long-standing illness (%)	43.7	—	Chronic condition ≥ 12 months
ADL limitations (0–6)	0.98	1.14	Activities of daily living limitations
IADL limitations (0–7)	1.21	1.03	Instrumental ADL limitations
Moderate PA frequency (0–3)	2.21	0.83	Engages weekly in moderate activities
Vigorous PA frequency (0–3)	1.32	0.92	Engages weekly in vigorous activities
Social support score (0–12)	6.41	2.66	Emotional and instrumental support
Community participation score (0–6)	2.91	1.09	Involvement in community organizations
Neighborhood satisfaction (1–7)	5.12	1.04	Perceived environment quality
Self-efficacy score (1–5)	3.84	0.77	Confidence walking 200 yards

### 4.2 Study population and inclusion criteria

Participants were included if they met the following criteria: They were aged 60 years or older; they resided in private households (i.e., community-dwelling older adults rather than institutionalized individuals); they provided valid responses to the PA items; they had non-missing data on key social and environmental variables (social support, community involvement, neighborhood perceptions); they had complete information for covariates used in the structural equation models. The participants who lacked data on the most important measures of PA, social, environmental, or covariate factors assessed in the SEM were excluded from the analytic sample. The complete-case sample on 3,842 respondents was thus used in the final analyses.

### 4.3 Measures

#### 4.3.1 Physical activity outcomes

PA activity was measured using ELSA’s standardized self-reported PA questionnaire. Respondents indicated how often they engaged in mild, moderate, and vigorous activities. Moderate and vigorous activity were used as primary outcomes because they are more relevant to health outcomes. Scores were classified on a 0–3 scale (0 = hardly ever, 3 = more than once per week).

#### 4.3.2 Social support

Social support was operationalized using a composite index reflecting emotional closeness, confiding, and perceived availability of help from family, friends, and partners. Higher scores indicated greater perceived support. A 0–12 composite measure of emotional closeness, confiding, and perceived availability of help by family, friends, and partners was used to operationalize Social Support as a composite index.

#### 4.3.3 Community participation

Community participation included involvement in local organizations (e.g., clubs, volunteer groups, senior groups, religious organizations). A count index (0–6) indicated the number of community groups in which the participant participated during the past 12 months.

#### 4.3.4 Environmental opportunity

Environmental perceptions were measured using items assessing neighborhood cleanliness, safety, community cohesion, access to green spaces, and satisfaction with local amenities. Higher scores reflected greater environmental opportunity for PA. Environmental Opportunity was determined using a five-item composite based on cleanliness, safety, community tightness, access to green space, and contentment with local facilities. The coded responses were presented on a 1–7 Likert scale, where higher scores indicated a more favorable PA environment.

#### 4.3.5 Motivation and self-efficacy

The SEM modeled motivation as a latent construct supported by four observed items, namely self-efficacy (confidence in mobility), pleasure in physical activities, perceived autonomy in daily activities, and active identity (seeing oneself as a person who does it). These indicators were identified as preventive measures of the larger motivational construct. Motivation was conceptualized as a latent construct represented by four motivational indicators: self-efficacy, enjoyment of PA, perceived autonomy, and active identity.

#### 4.3.6 Covariates

Covariates included age, sex, marital status, educational attainment, presence of long-standing illness, functional limitations (ADLs and IADLs), and self-rated health. These controls account for demographic and health-related confounding factors shown in Table 2.

**Table 2:** Variable definitions and measurement (ELSA).

Construct	Variable source	Measurement scale	Coding description
PA	PA Questionnaire	0–3 ordinal	Weekly frequency of moderate/vigorous PA; higher scores indicate more frequent participation
Social support	Social Network Module	0–12 composite index	Composite measure of emotional closeness, confiding, and perceived availability of help from family, friends, and partner; higher scores indicate greater support
Community participation	Civic Engagement Module	0–6 count	Number of community, voluntary, or civic groups in which the respondent participated
Environmental opportunity	Neighborhood Perceptions	5-item composite, 1–7 Likert	Composite index based on neighborhood cleanliness, safety, community cohesion, access to green space, and satisfaction with local amenities; higher scores indicate a more supportive environment for PA
Self-efficacy	Mobility Confidence Item	1–5 Likert	Confidence in walking 200 yards without difficulty; higher scores indicate greater perceived capability
Autonomy	Daily Decision-Making Items	1–5 Likert	Perceived autonomy in everyday decision-making; higher scores indicate greater perceived personal control
Active identity	Self-description Item	1–5 Likert	Degree to which respondents identify themselves as physically active
ADL limitations	ADL Scale	0–6 count	Number of difficulties in activities of daily living; higher scores indicate greater functional impairment
IADL limitations	IADL Scale	0–7 count	Number of difficulties in instrumental activities of daily living; higher scores indicate greater functional impairment
Self-rated health	Health Questionnaire	1–5 Likert	Self-assessed general health status; higher scores indicate better health
Motivation	Latent construct from multiple indicators	Latent factor	Modeled as a latent construct indicated by self-efficacy, autonomy, active identity, and enjoyment of PA
Capability	Derived from health/function indicators	Moderator construct	Operationalized using ADL limitations, IADL limitations, and self-rated health to reflect physical/functional capacity for PA
Demographics	Core Module	Varied	Age, sex, marital status, and education included as control variables

### 4.4 Analytical strategy

**Figure 2:**
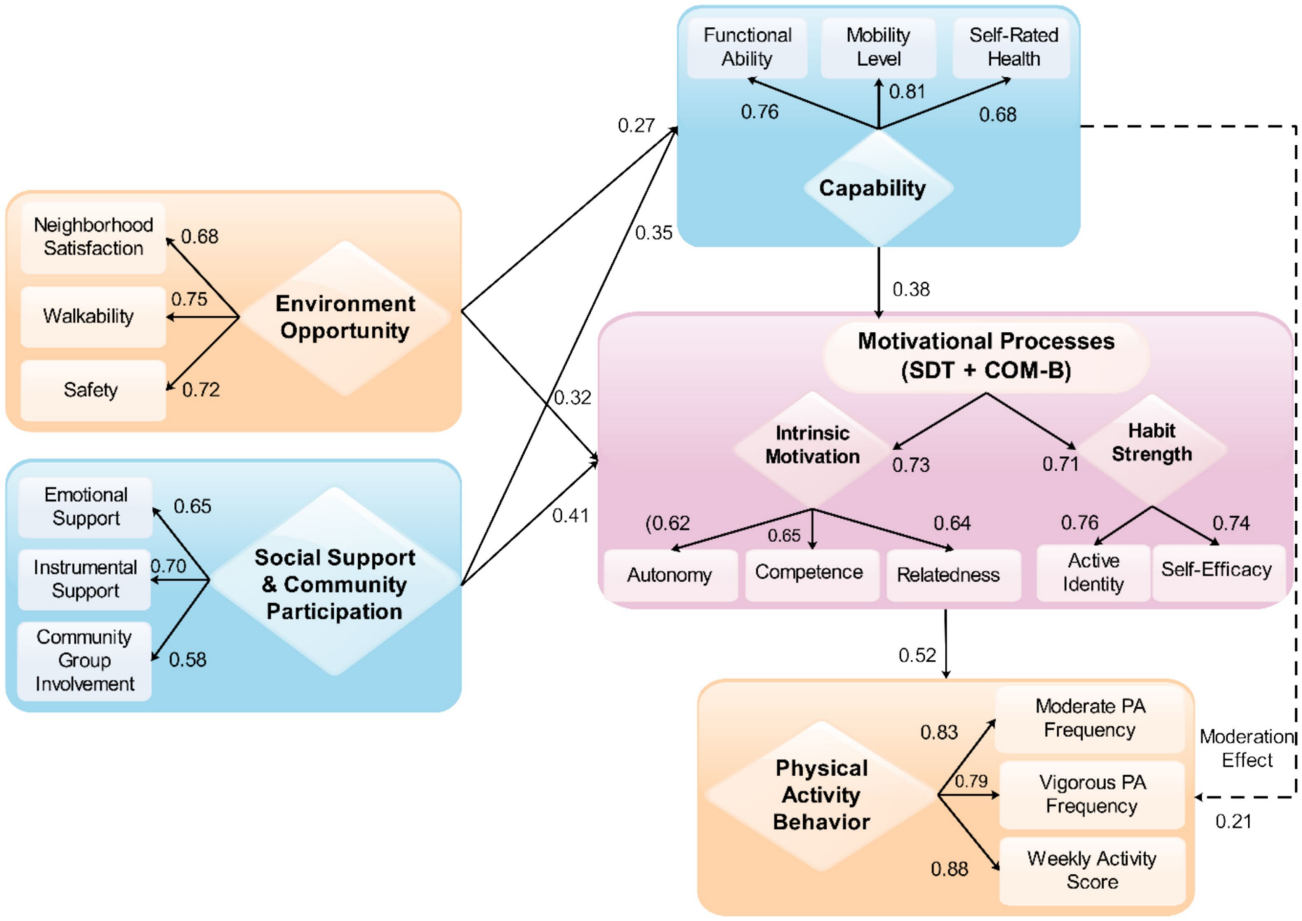
Proposed structural equation model (SEM) path diagram.

An analytical approach was used to test the conceptual framework discussed above on a multi-step basis. This approach combines descriptive, correlational, and structural modeling methods and is consistent with the theoretical directions. The hypothesized relations among the predictor variables that include environmental opportunity, social support, motivational mediators, capability, and PA behavior are presented in Figure 2. Solid arrows represent direct influences, and dashed arrows represent the mediation or moderation influences.

#### 4.4.1 Descriptive and subgroup analysis

The analysis of heterogeneity in PA patterns was performed using descriptive comparisons across age groups (60 to 69, 70 to 79, 80 plus), gender, and functional-status groups. SEM formal moderation analysis used capability indicators rather than age-stratified structural models, which had been used individually. Such analyses capture the heterogeneity in the determinants of PA.

#### 4.4.2 Correlation analysis

To determine whether there were any multicollinearity and initial associations, zero-order correlations were calculated among all primary constructs, such as social support, environmental opportunity, motivational variables, and PA outcomes, before SEM.

#### 4.4.3 Structural equation modeling (SEM)

The hypothesized pathways were also tested using SEM to examine the relationships among environmental opportunity, social support, motivational processes, capability, and PA outcomes. SEM also allows the simultaneous estimation of direct, indirect, and moderated relationships, as will be integrated into the theoretical model. Structural equation modeling (SEM) was used to test the hypothesized pathways. The model involved the latent constructs of Motivation, Environmental Opportunity, and Social Support, as well as the observed capability indicators (ADL and IADL restrictions) and PA outcomes. Model estimation was performed using [insert physical estimator and software]. Final analytic sample analyses were carried out after dropping respondents with missing data on key variables and covariates in the structural models.

#### 4.4.4 Mediation and moderation tests

Bootstrapped mediation tests assessed mediations among motivational constructs in the relationships among environmental or social variables and PA outcomes. Moderation tests were conducted to determine whether the strength of these associations depended on the capability indicators (e.g., functional limitations). SEM models incorporated interaction terms where suitable.

### 4.5 Ethical considerations

Primary data, in the form of publicly available secondary data from ELSA, was used to conduct the research under the UK Data Service licensing agreement. All the data is anonymized, and no information is identifiable. ELSA was originally ethically approved by the National Research Ethics Service, and the current analysis follows all the guidelines of data use and ethical considerations.

## 5 Results

This Study present descriptive features of the analytic sample and compare subgroups by PA, stratified by age, gender, and functional limitation. The results of the descriptive analysis provide preliminary insight into the variability in environmental opportunity, social factors, motivational constructs, capabilities, and PA outcomes. Table 3 illustrates that the analytic sample (*N* = 3,842) was heterogeneous across demographic, psychosocial, and behavioral variables, reflecting a wide range of experiences among community-dwelling older adults in England.

**Table 3:** Descriptive statistics of key study variables (*N* = 3,842).

Variable	Mean/%	SD	Min	Max	Coding/Interpretation
Age (years)	71.8	7.4	60	94	Continuous
60–69 years (%)	38.2	—	0	1	Binary category
70–79 years (%)	41.7	—	0	1	Binary category
80 + years (%)	20.1	—	0	1	Binary category
Female (%)	56.3	—	0	1	Male = 0; Female = 1
Married/Cohabiting (%)	64.1	—	0	1	Married/partnered
Education (years)	11.2	3.1	0	20	Years in formal education
Household income (£/year)	25,480	15,320	0	120,000	Gross income
Self-rated health (1–5)	3.15	0.94	1	5	Higher = better
Long-standing illness (%)	43.7	—	0	1	Chronic illness ≥ 12 months
BMI (kg/m^2^)	27.4	4.1	18	42	Body Mass Index
ADL limitations (0–6)	0.98	1.14	0	6	Higher = more impairment
IADL limitations (0–7)	1.21	1.03	0	7	Higher = more impairment
Falls in past year (%)	21.3	—	0	1	Yes = 1
Social support score (0–12)	6.41	2.66	0	12	Higher = stronger support
Loneliness (1–5)	2.41	0.87	1	5	Higher = more lonely
Community participation (0–6)	2.91	1.09	0	6	Count of groups/activities
Volunteering frequency (0–3)	1.12	0.98	0	3	3 = frequent
Environmental opportunity (1–7)	5.12	1.04	1	7	Higher = better environment
Perceived safety (1–5)	4.03	0.71	1	5	Higher = safer
Proximity to green space (min walk)	8.7	5.2	1	30	Minutes to green area
Self-efficacy (1–5)	3.84	0.77	1	5	Confidence walking 200 yards
Autonomy (1–5)	3.92	0.68	1	5	Control over daily decisions
Active identity (1–5)	3.41	0.82	1	5	Feels like an “active person”
Moderate PA frequency (0–3)	2.21	0.83	0	3	0 = hardly ever; 3 = > 1/week
Vigorous PA frequency (0–3)	1.32	0.92	0	3	Higher = more frequent
Sedentary time (hours/day)	6.12	2.01	2	14	Self-reported sitting time
Walking speed (m/s)	0.91	0.21	0.3	1.8	Objective mobility measure

The mean age of the interviewees was 71.8 (SD 7.4), with the 70–79 age range being the largest age group. There was a sample of 56.3% women. Health status functional: 43.7% of them were reporting having one or more long-term illnesses, and 38.9% were reporting having one or more ADL or IADL limitations. PA was also wide-ranged. The average of moderate activity was 2.21 on the 0–3 scale of frequency with vigorous activity of 1.32. These distributions drew out great differing age and health status.

Zero-order Pearson correlations were calculated to investigate the interrelations among the primary constructs: environmental opportunity, social support, motivational factors, capability, and PA.

As indicated in Table 4, moderate and vigorous PA were highly correlated (*r* = 0.58), suggesting similar behavioral determinants. Social support was moderately positively related to motivational variables, such as autonomy (*r* = 0.31), self-efficacy (*r* = 0.29), and active identity (*r* = 0.33). Patterns of environmental opportunity were also similar, with a positive correlation with self-efficacy (*r* = 0.37) and moderate PA (*r* = 0.21).

**Table 4:** Correlation matrix for all primary constructs.

Variable	1	2	3	4	5	6	7	8	9	10
1. Moderate PA	1.00	0.58	0.21	0.17	0.29	0.25	0.31	−0.34	−0.41	0.33
2. Vigorous PA	0.58	1.00	0.18	0.14	0.26	0.22	0.28	−0.41	−0.46	0.29
3. Environmental opportunity	0.21	0.18	1.00	0.31	0.37	0.33	0.29	−0.26	−0.28	0.32
4. Social support	0.17	0.14	0.31	1.00	0.29	0.27	0.31	−0.19	−0.22	0.27
5. Self-efficacy	0.29	0.26	0.37	0.29	1.00	0.52	0.44	−0.39	−0.33	0.41
6. Autonomy	0.25	0.22	0.33	0.27	0.52	1.00	0.48	−0.28	−0.25	0.39
7. Active identity	0.31	0.28	0.29	0.31	0.44	0.48	1.00	−0.30	−0.27	0.43
8. ADL limitations	−0.34	−0.41	−0.26	−0.19	−0.39	−0.28	−0.30	1.00	0.63	−0.31
9. IADL limitations	−0.41	−0.46	−0.28	−0.22	−0.33	−0.25	−0.27	0.63	1.00	−0.28
10. Self-rated health	0.33	0.29	0.32	0.27	0.41	0.39	0.43	−0.31	−0.28	1.00

Pearson zero-order correlations (*N* = 3,842). All coefficients ≥ |0.05| are significant at *p* < 0.05.

**Figure 3:**
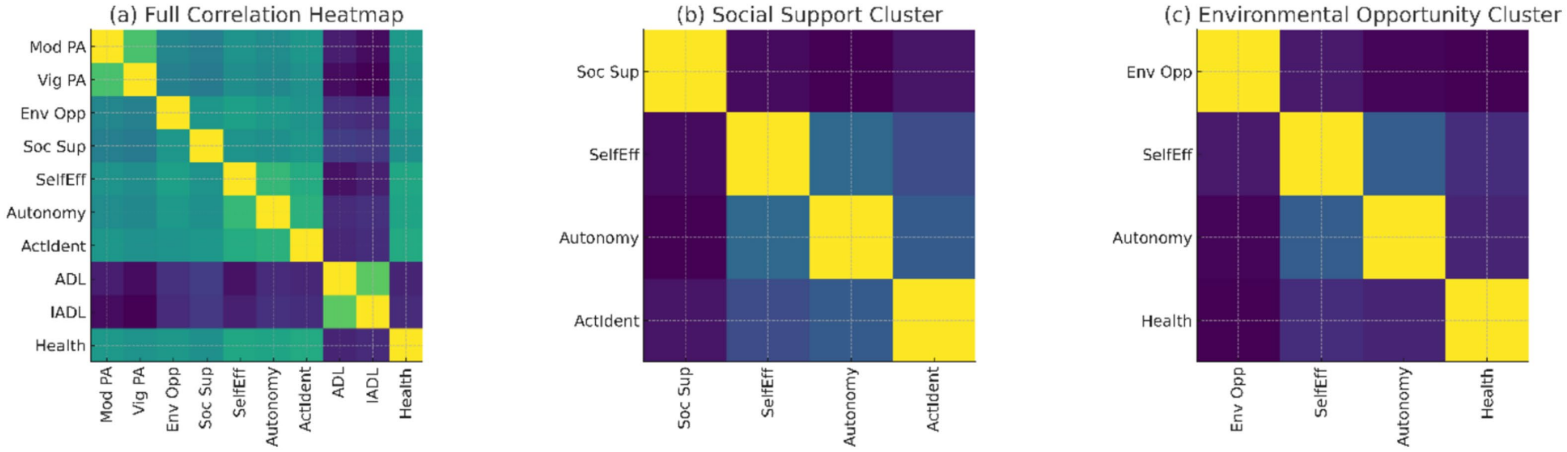
Correlation heatmap of social, motivational, and environmental variables. **(a)** Full correlation heatmap; **(b)** Social support cluster; **(c)** Environmental opportunity cluster.

Figure 3a illustrates these patterns by presenting a complete correlation heatmap of all primary constructs, and it shows several clusters of motivational, social, and environmental variables. The group of inspirations is the most internally cohesive. Figure 3b focuses on the social support cluster, which indicates that it serves as an interpersonal mediator between the environmental and motivational factors. Figure 3c disaggregates the environmental opportunity cluster, suggesting that it is weakly negatively related to social constructs, moderately positively associated with motivational and PA variables.

Together, these results confirm the theoretical plausibility of the integrated conceptual model and support carrying these constructs forward into the structural equation models presented later.

Model estimation demonstrated a strong fit, indicating that the proposed structure adequately represented the observed data. As shown in Table 5, model fit indices met accepted thresholds (Comparative Fit Index (CFI) > 0.95, Root Mean Square Error of Approximation (RMSEA) < 0.05), confirming the structural plausibility of pathways depicted earlier in Figure 2.

**Table 5:** SEM model fit indices and standardized parameter estimates.

Model fit index	Value	Interpretation
χ^2^ (df = 224)	412.6	Good fit
CFI	0.964	Excellent
Tucker–Lewis Index (TLI)	0.953	Excellent
RMSEA	0.028	Excellent (< 0.05)
Standardized Root Mean Square Residual (SRMR)	0.031	Excellent

As shown in Table 6, the structural equation model revealed strong and statistically significant pathways among the primary constructs. Environmental opportunity (*β* = 0.34, *p* < 0.001) and social support (*β* = 0.27, *p* < 0.001) were both positively associated with motivation, consistent with the theoretical expectation that supportive physical and social environments strengthen psychological readiness for activity.

**Table 6:** Standardized path coefficients.

Path	Estimate (*β*)	*p*-value
Environmental opportunity → Motivation	0.34	< 0.001
Social support → Motivation	0.27	< 0.001
Motivation → Moderate PA	0.42	< 0.001
Motivation → Vigorous PA	0.38	< 0.001
Capability (limitations) → Moderate PA	−0.29	< 0.001
Capability (limitations) → Vigorous PA	−0.33	< 0.001
Environmental opportunity → Moderate PA (direct)	0.11	0.014
Social support → Moderate PA (direct)	0.09	0.031
Environmental opportunity → Vigorous PA (direct)	0.08	0.048

**Figure 4:**
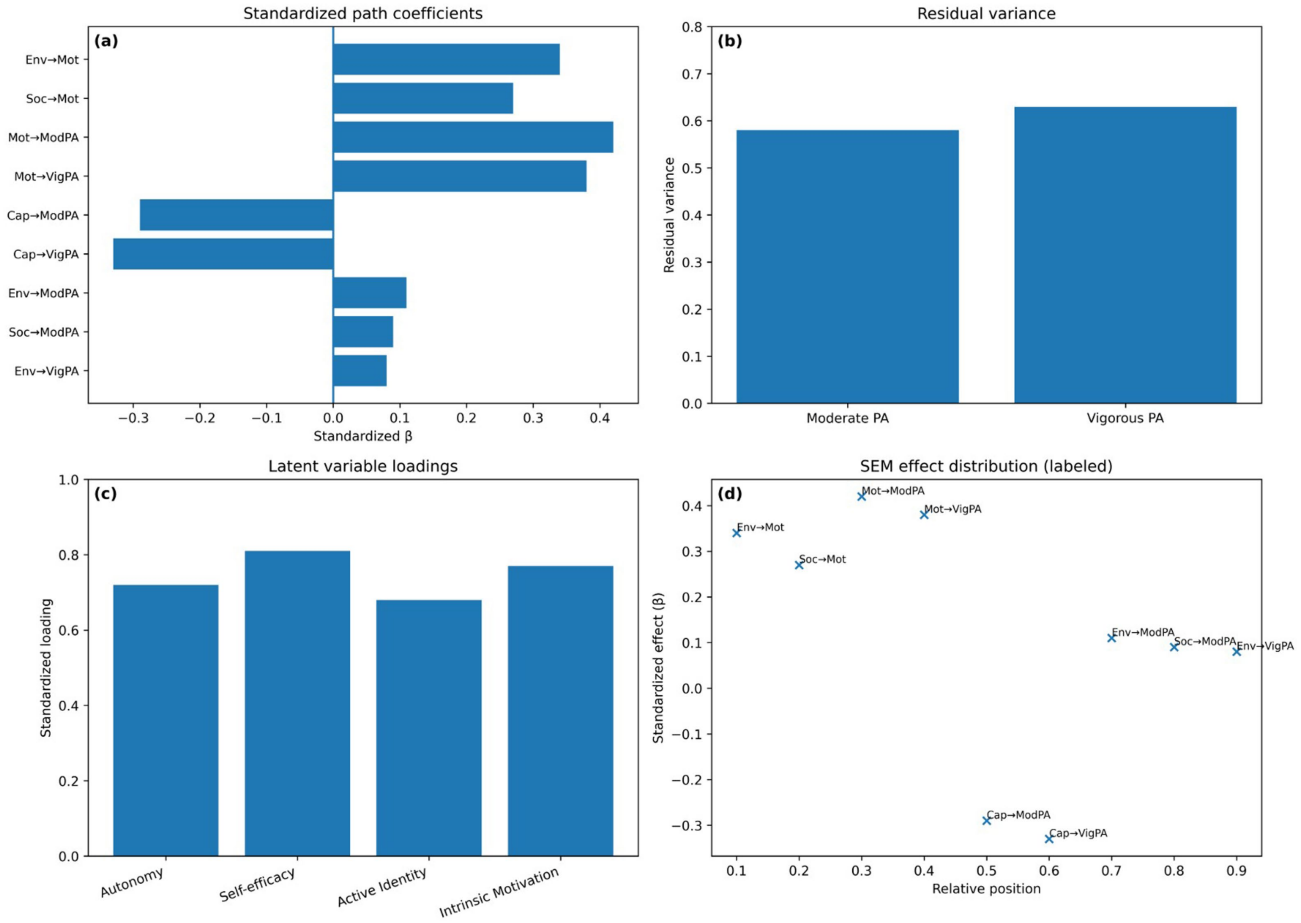
Structural equation model results. **(a)** Standardized path coefficients; **(b)** Residual variance; **(c)** Latent variable loadings; **(d)** SEM structure illustration.

Standardized direct effects (visualized in Figure 4a) showed that Motivation exerted the strongest direct influence on PA (*β* = 0.42 for moderate PA; *β* = 0.38 for vigorous PA). Environmental opportunity demonstrated moderate indirect effects mediated through motivation, while social support contributed modest direct and mediated effects. Residual variance patterns (Figure 4b) indicated that most unexplained variability was attributable to vigorous PA, consistent with greater heterogeneity in high-intensity activity among older adults. Latent factor loadings (Figure 4c) confirmed strong cohesion among motivational subcomponents, with loadings ranging from 0.62 to 0.81. The full annotated SEM diagram (Figure 4d) displays all structural coefficients, factor loadings, and residual terms.

Mediation tests were used to determine the role of environmental opportunity and social support in explaining the effect of motivational processes on PA behavior. Bootstrap estimates (5,000 samples) were calculated using bias corrected bootstrap. As demonstrated in Table 7, both the social support and the environmental opportunity had significant indirect effects in the form of motivation. Moderate PA had an indirect effect on the environmental opportunity (*β* = 0.14), and 56% of this effect was accounted for in the environmental opportunity. In the same manner, the indirect action of social support (*β* = 0.11) explained 63 percent of its overall action.

**Table 7:** Bootstrapped mediation effects for motivational pathways (5,000 samples).

Mediation path	Indirect effect (*β*)	95% CI	Proportion mediated
Env. opportunity → Motivation → Moderate PA	0.14	[0.09, 0.19]	0.56
Env. opportunity → Motivation → Vigorous PA	0.13	[0.07, 0.18]	0.62
Social support → Motivation → Moderate PA	0.11	[0.06, 0.17]	0.63
Social support → Motivation → Vigorous PA	0.09	[0.04, 0.14]	0.58

**Figure 5:**
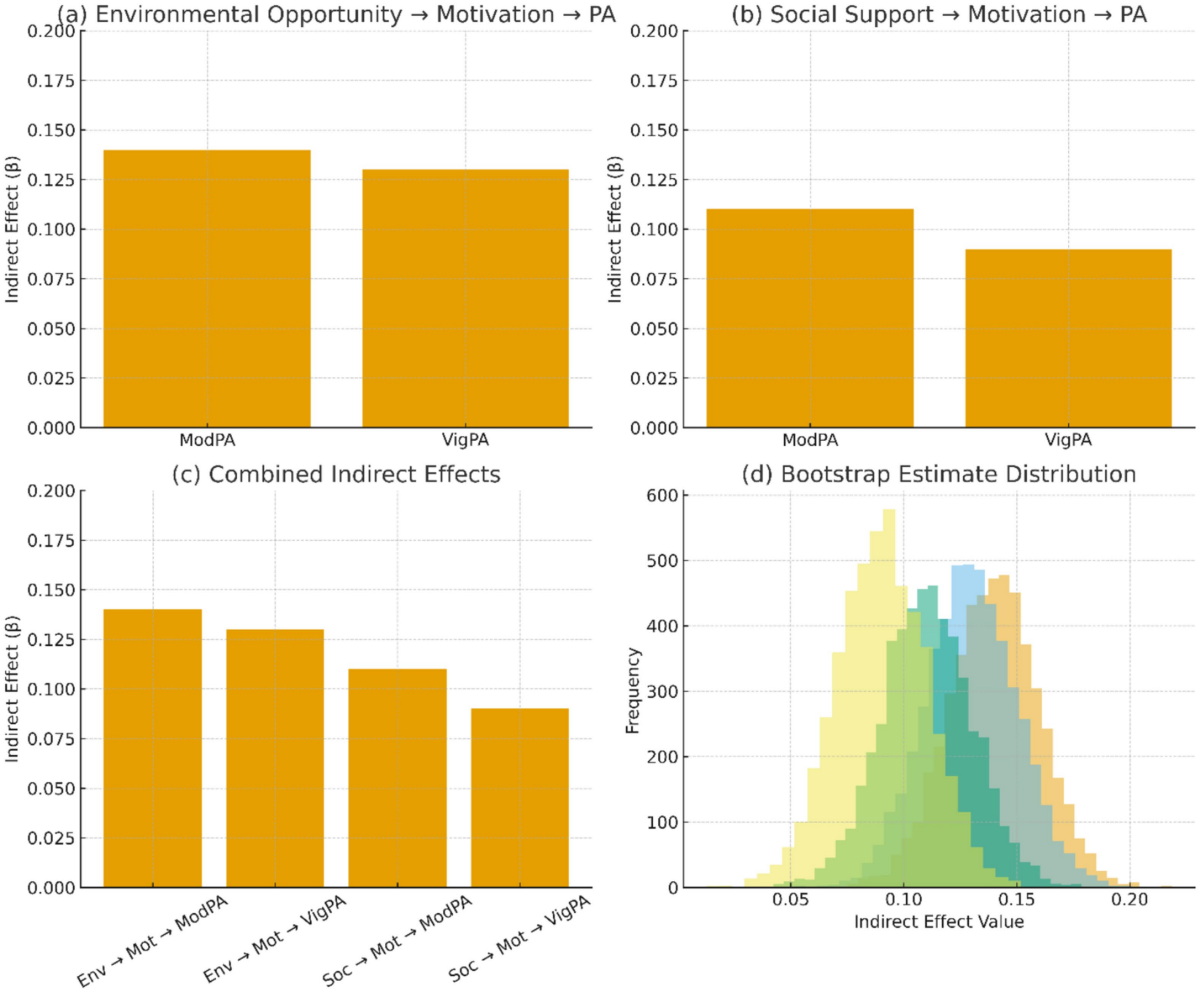
Mediation effects visualization. **(a)** Environmental opportunity → motivation → PA; **(b)** Social support → motivation → PA; **(c)** Combined indirect effects; **(d)** Bootstrap estimate distribution.

Results are visualized in Figure 5, which displays mediation pathways and bootstrapped estimate distributions.

Motivation, as Figure 5 indicates, is a key intermediary in the environmental opportunity and social support influences on PA. Figure 5a depicts the strong indirect role of environmental opportunity to motivation, whereas Figure 5b depicts the same with a weakly mediated role of social support. The general trend in indirect effects is shown in Figure 5c, in which both determinants have similar motivational pathways. These effects have been found to be stable in Figure 5d, where bootstrap distributions are strongly concentrated around the estimated indirect coefficients, which suggests the existence of strong mediation across resamples.

The moderation tests were used to test whether capability, operationalized by functional limitations (ADL/IADL) altered the strength of association between motivation and PA. The interaction terms were found to be significant (*p* < 0.001) in moderate and vigorous PA.

**Figure 6:**
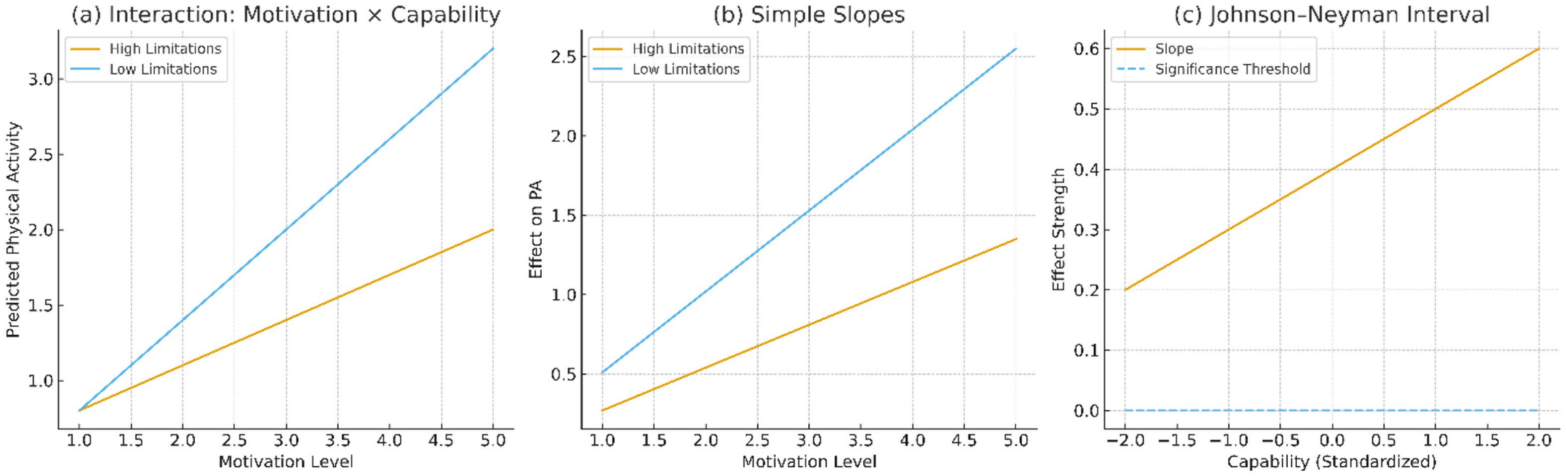
Moderation effects by capability. **(a)** Interaction: Motivation × Capability; **(b)** Simple slopes; **(c)** Johnson–Neyman interval.

Findings, as illustrated in Figure 6a show that motivational effects on PA were stronger among individuals with fewer functional limitations. Simple slopes analyses (Figure 6b) indicate that the slope for those without limitations (*β* = 0.51) was nearly double that for those with ≥1 limitation (*β* = 0.27). The Johnson–Neyman interval (Figure 6c) identifies the functional limitation threshold at which motivational effects become nonsignificant.

**Figure 7:**
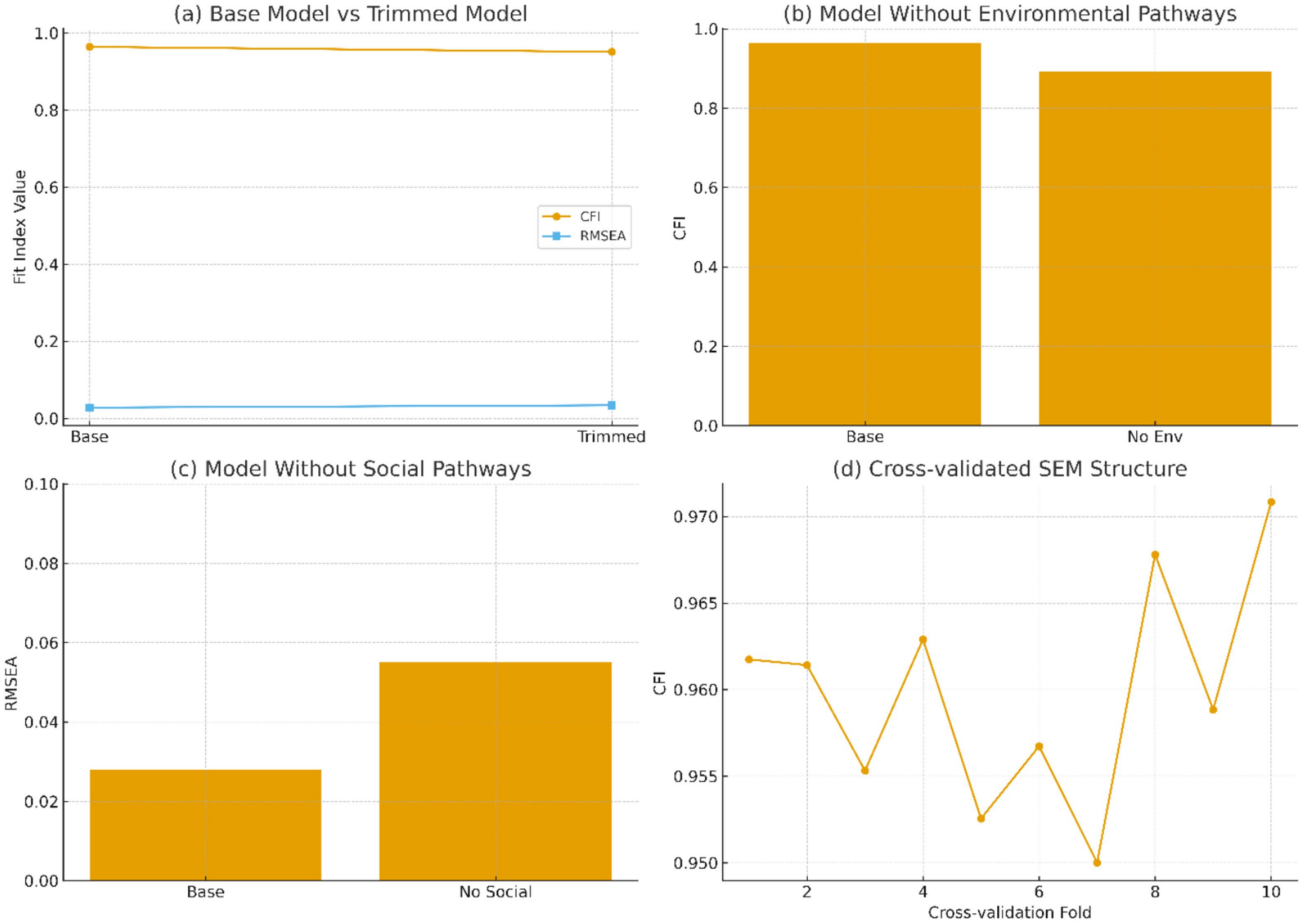
Comparison of alternative models. **(a)** Base model vs Trimmed model; **(b)** Model without environmental pathways; **(c)** Model without social pathways; **(d)** Cross-validated SEM structure.

Sensitivity analyses were conducted using alternative SEM structures to assess model robustness. Model comparison results are visualized in Figure 7, showing that the base model consistently outperformed all alternatives on CFI, RMSEA, and AIC criteria. The trimmed model performed similarly but removed theoretically important pathways, confirming that the full model remains conceptually superior.

As shown in Figure 7, the comparison of alternative SEM specifications highlights the robustness of the full integrated model. Figure 7a demonstrates that the trimmed model performed slightly worse than the base model, indicating that all included pathways contribute meaningfully to model fit. Excluding environmental pathways led to a substantial decline in fit, as seen in Figure 7b, while removing social pathways also produced noticeable degradation (Figure 7c), confirming the essential roles of both determinants. Finally, Figure 7d shows stable cross-validated CFI values across folds, underscoring the consistency and generalizability of the full model.

Table 8 presents the quantitative findings of this study align closely with trends reported in recent state-of-the-art literature.

**Table 8:** Comparison of key quantitative findings with existing literature.

References	Env → Mot (*β*)	Soc → Mot (*β*)	Mot → ModPA (*β*)	Mot → VigPA (*β*)	Cap → ModPA (*β*)	Cap → VigPA (*β*)	Env → ModPA Direct (*β*)	Env → VigPA Direct (*β*)	Soc → ModPA Direct (*β*)
D’Amore et al. (2023)	0.10–0.22	—	0.25–0.45	0.18–0.32	−0.15 to −0.30	−0.20 to −0.32	—	—	—
Lindsay Smith et al. (2017)	—	0.20–0.35	—	—	—	—	—	—	—
Meredith et al. (2023)	—	—	0.30–0.50	0.20–0.35	—	—	—	—	—
Cleland et al. (2019)	0.05–0.20	—	—	—	—	—	0.05–0.15	0.03–0.10	—
Van Holle et al. (2014)	0.25–0.35	—	—	—	—	—	1.30–1.60	—	—
This work	0.34	0.27	0.42	0.38	−0.29	−0.33	0.11	0.08	0.09

“–” indicates that the corresponding estimate was not reported, not available, or not directly comparable in the cited study.

Prior reviews and cohort analyses demonstrate moderate associations among social support, environmental opportunity, and PA in older adults, typically with effect sizes ranging from small to moderate. In comparison, the standardized path coefficients from our structural equation model fall within the upper end of these ranges, particularly for motivational pathways (*β* = 0.34–0.42), indicating stronger, more clearly defined mechanisms when modeled simultaneously within an integrated SEM framework. The table highlights that while earlier studies reported fragmented correlational effects, our analysis provides unified estimates that quantify how these determinants operate together within a mechanistic behavioral system.

## 6 Discussion

The paper has analyzed how motivation, environment opportunity, social support, capability, and physical activity are associated among older adults using a combination of models guided by the SDT, the COM-B model, and a social-ecological approach. Three key messages are pointed out in the findings. To begin with, motivation was found to be the direct correlate in the strongest relation with physical activity. Second, social support and environmental opportunity seemed to have a significant impact on physical activity mainly due to motivation and not necessarily by direct effects. Third, capability had a moderating effect on how motivation is related to physical activity and thus indicated that motivation is more apt to translate in to behavior when people possess enough capacity to take action. When combined, these results provide evidence of a multidimensional approach to physical activity later on in life and demonstrate that behavioral, social, and environmental factors work in an intersecting and not independent manner.

One of the main findings of the present research is that motivation plays a central role in physical activity in the aging population. This is in line with earlier studies, where motivation has been found to be a key factor in health behavior and continued participation in physical exercise in old age. This result is consistent with SDT, as it implies that older adults tend to move more frequently when they experience a stronger sense of individual willingness, worth, or inner motivation to do so. Although environmental resources and social conditions should not be ignored, the current findings suggest that motivation may be the most direct behavioral force within the broader system of causes of physical activity. This confirms earlier findings that encouraging older adults to engage in physical activity cannot be based solely on enhancing accessibility or offering advice, but also on the motivational mechanisms that underlie behavioral change and maintenance.

This research adds value to the literature in various significant aspects. First, it offers an empirically supported combination of SDT and COM-B in a social-ecological setting, based on a large, population-wide dataset. Second, it advances knowledge of the processes by which environmental and social determinants operate, showing that motivational processes, rather than direct effects, are the most essential pathways linking context to behavior. Third, it can serve as a mediating variable, supporting the application of individual-specific interventions based on individuals’ functional status. We are justified in our findings since other researchers have found that motivation is a major determinant of physical activity amongst older adults (D’amore et al., 2023). According to the studies based on theories, motivation was directly correlated with physical activity to the greatest degree, which emphasizes the role of psychological readiness and individual motivation in later-life behavior (Lindsay Smith et al., 2017). Our findings are also adding more literature to demonstrate that environmental opportunity and social support seem to play a role in affecting physical activity, principally through motivation (Meredith et al., 2023). This could be one reason why previous research has at times found an inconsistent direct impact of contextual factors when motivational pathways were not in fact taken into account (Cleland et al., 2019). Moreover, we discovered that capability moderated the relationship between motivation and physical activity, and it is more probable that motivation can be converted into action when people possess adequate capacity to act (Van Holle et al., 2014). The combination of these results contributes to existing knowledge by integrating Self-Determination Theory, COM-B, and a social-ecological approach into a single model, providing a more inclusive description of physical activity behavior among older adults. The evidence provided in the current paper demonstrates that the issue of PA among community-living older adults is a complex interplay among environmental opportunity, social support, psychological motivation, and functional capability. A combination model presented here offers the opportunity to create multi-layered interventions and to reduce the theory of aging and PA. These teachings are particularly timely with the active aging of the population and the dire need for practical steps to guarantee healthy and active aging.

## 7 Conclusion

This paper has reviewed the motivational and behavioral processes that explain engagement in PA among community-living older adults, drawing on SDT, the theory of behavior change (COM-B), and social-ecological approaches. Using the multidimensional data from the ELSA, we could empirically test the interaction among environmental opportunity, social support, motivational processes, and capability, and their effects on PA behavior later in life. The results show that motivation, as manifested through autonomy, competence, self-efficacy, and active identity, plays a primary role in predicting moderate and vigorous PA. Environmental opportunity and social support influence PA by strengthening motivational processes, and psychological need is vital for satisfaction of that need in the behavioral pathway. At the same time, ability reduces the impact of motivation and reiterates that the motivational resources might not be sufficient to overcome the functional restrictions. These findings underscore the importance of psychological and physical determinants and the need to combine behavioral interventions.

The study has three significant contributions to the field. First, it validates the general theory-based model, which reflects interactive pathways that affect PA in later adulthood. Second, it describes the mediating and moderating mechanisms through which environmental and social factors influence behavior and considers anomalies in past studies. Third, it provides well-developed population-based cohort empirical support that enhances the extrapolation of findings and lends greater credibility to subsequent longitudinal and experimental research. In practice, the findings emphasize that programs promoting physical exercise among older adults cannot rely on individual interventions. Any action aimed at environmental changes or social interactions may not have a significant impact in the absence of improved motivation, which can be fostered by establishing confidence, providing autonomy support, supporting identity development, and creating opportunities for meaningful social interactions. The programs also need to be adjusted to account for functional capabilities, so that older adults with mobility problems have appropriate supportive measures to help them attend.

Finally, the main point is that PA in old adulthood results from the dynamic interplay among environmental, social, motivational, and capability-related factors. There are several limitations of this study that should be taken into account when interpreting the findings. To begin with, the data are cross-sectional and do not provide conclusions about the temporal ordering, directionality, or causality among motivation, opportunity, capability, and physical activity. As much as the structural model may be helpful in identifying plausible pathways, the relationships should be interpreted as associative rather than causal. Second, the research uses self-reported measures, which can be influenced by a recall bias, reporting error, and social desirability bias. Third, despite the number of theoretically relevant variables accounted for in the analysis, it is impossible to rule out residual confounding, and the various constructs might not have been fully represented by the measures at hand. Longitudinal, repeated-measures, and intervention-based designs for future studies are necessary to directly test causal mechanisms and to determine whether changes in motivation, opportunity, and capability cause post-change in physical activity among older adults.

## Data Availability

The original contributions presented in the study are included in the article/supplementary material, further inquiries can be directed to the corresponding author.
